# A Review of the Urban Development and Transport Impacts on Public Health with Particular Reference to Australia: Trans-Disciplinary Research Teams and Some Research Gaps

**DOI:** 10.3390/ijerph6051557

**Published:** 2009-04-28

**Authors:** Deborah Black, John Black

**Affiliations:** 1 Health Informatics and Statistics Research Group, Faculty of Health Sciences, T Block Room 310, Cumberland Campus, University of Sydney, NSW 2006, Australia; E-Mail: deborahblack@usyd.edu.au; 2 Center for North East Asian Studies, Tohoku University, 41 Kawauchi, Aoba-ku, Sendai, 980-8576, Japan; and School of Civil and Environmental Engineering, The University of New South Wales, NSW 2052, Australia

**Keywords:** Multi-disciplinary teams, urbanization and transport, environmental stressors, public health, research and policy interventions

## Abstract

Urbanization and transport have a direct effect on public health. A transdisciplinary approach is proposed and illustrated to tackle the general problem of these environmental stressors and public health. Processes driving urban development and environmental stressors are identified. Urbanization, transport and public health literature is reviewed and environmental stressors are classified into their impacts and which group is affected, the geographical scale and potential inventions. Climate change and health impacts are identified as a research theme. From an Australian perspective, further areas for research are identified.

## 1. Introduction

Health can be viewed as a central criterion for judging human sustainability [1]. A complete understanding of this dimension of human health requires knowledge about the effects of global economic and climate change on: ecosystem sustainability and on human health; on the effects of pollutants within human communities; on the interaction between environment, development, and human health; and on the management of solutions to these challenges across local, regional, and global scales. Against this perspective on health and sustainability the scope of this review is restricted to urban development and transport as potential environmental stressors. Within this narrowed scope it is therefore economic development (more specifically, urbanization and transport infrastructure development) that can impair public health if environmental and social considerations are neglected, however, we do consider global climate change as a driver factor in shaping future environmental stressors in the city.

Cities are significant “places” to analyze environmental stressors and public health problems. The early 21st century was marked by the extraordinary fact that for the first time in history more people on the planet live in urbanized areas than in rural ones. The geographical focus of this article is on urban settlements. In the developing world, the hinterlands around the urbanized areas have their own distinctive problems, associated partly with transport and communication: health problems are tied to poverty and to isolation and lack of access (see, for example [2], for the specific case of Laos). These problems differ substantially from the health issues in the rapidly expanding cities of the developing world. Within any one human settlement, and especially in the larger metropolitan regions, environmental stressors clearly have both location and time dimensions (duration by time of day, seasonality, trends over time), and it is precisely these aspects that we conceptualize in Section 3 and indicate research gaps, including the difficult empirical analyzes to account for a person’s life-time exposure to environmental stressors (Section 4).

As humans alter the character of the natural landscape of any region in the urbanization process (the driving forces of change), they directly influence the magnitude of environmental stressors such as impact on regional air quality, energy consumption, and local and regional, and global scale climates. Urban re-development results in changes in land uses, with their associated, more intensive, economic and social activities and these have an additional impact on environmental stressors. These various aspects of the urbanization process impact on human health. It is the complexity of these emerging public health problems that present a major new challenge for sustainable development, well-being and the quality of life in cities. Integrated solutions will require health care professionals, epidemiologists, engineers, environmental scientists, urban planners, designers and managers, policy specialists, economists and social scientists to find new ways to work. The trans-disciplinary approach offers a promising organizational framework. Therefore, in Section 2, “team science” is required to frame the issues, to undertake analyzes of the complex interactions between urban form, transport and health and to generate specific solutions of mitigation and adaptation. A critical case study of the interdisciplinary approach is presented given the recent interest in the science of “team science.”

In Section 3, we present a descriptive, conceptual model of the characteristics of a hypothetical region that give rise to environmental stressors impacting on health. The model considers the causal relationship between human activity, the pressures they place on the environment, the feedback from those activities on people in the form of environmental pressures, and the actions that are taken by governments, businesses and society in response to these pressures. In the approach taken with the descriptive model we use the typical language of state of the environment reporting: **driving** forces of environmental change; **pressures** on the environment; the **state** of the environment; **impacts** on the population; and the **response** of the society.

Section 4 contains the results of our literature review of publications across the fields of urban form, transport and public health, where the evidence suggests there are quite distinctive outputs of scientific research across these themes. The classification of this diffuse literature is summarized with particular emphasis on the demographic and socio-economic characteristics of those affected, on the location and geographical scale of the environmental stressor, and on the broad categories of types of policies, programs and solutions. The main research gaps in the literature can be summarized as a need to introduce the long-term temporal dynamics into research investigations including the diurnal time-dependent nature of some stressors and the life histories of individuals given that as bodies age exposures over a life time of environmental stressors can accelerate the aging process and trigger disease. These research gaps relate to Australia where an identification of trans-disciplinary research centers and work in progress has lead to the particular research suggested. Although these are country-specific suggestions they probably have relevance to other countries, noting that Australia is a liberal democratic country and therefore suggested methodologies and solutions may not be transferable to many parts of the urbanized world.

## 2. Trans-disciplinary Research Teams

Improved transport links have both encouraged rural-urban migration flows and have provided the means for urban dwellers to access the land-use activities contained within those urban regions. It is the scale and concentration of these human activities that generate environmental stressors. Cities are objects, which by definition, pertain to many realities and are studied by numerous disciplines [3]. In the transport discipline, the premier international research body – the World Conference on Transport Research Society (WCTRS) – is represented by many disciplines drawn from academia, the professions and policy makers. The scientific society is structured with approved Special Interest Groups, including transport and the environment, which aims at seeking ways to establish effective mechanisms for mitigating environmental degradation due to transport in the international domain [4], yet “transport and health” is yet to be nominated as a sub-theme. The general experience is that interdisciplinary, or multi-disciplinary, team science is being more important, especially through international collaborative exercises but the trans-disciplinary approach is limited.

The public health field also has its similar weaknesses of inter-connections with other disciplines. Despite the last decade of the twentieth century witnessing a profusion of projects drawing together multi-disciplinary teams of social and health scientists to study and recommend solutions for a wide range of health problems, a trans-disciplinary approach is required to provide a systematic, comprehensive theoretical framework for the definition and analysis of the social, economic, political, environmental, and institutional factors influencing human health and well-being [5]. Team research is expected to continue its dominance in the production of knowledge in the 21^st^ century. A transdisciplinary research approach will become more firmly entrenched as the preferred methodology on major research investigations. For example, the mission of the International Association for Ecology and Health (EcoHealth) is to strive for the sustainable health of people, wildlife, and ecosystems. Trans-disciplinary pursuits involve combining strengths to achieve hybridized innovative approaches to problem-solving. Such challenges demand this level of strategic team-based approaches to the tightly coupled human-natural system interactions underlying many of the pressing threats to our sustained health and environment.

This prediction about the trans-disciplinary approach is based on an interpretation of the analysis of 19.9 million research articles in the Institute for Scientific Information (ISI) Web of Science database and an additional 2.1 million patent records [6]. There has been a steady increase in team size over the last five decades, and teams now dominate the top of the citation distribution in all four research domains of sciences and engineering, social sciences, humanities, and patents. Even proponents of team science initiatives note that they are highly labor intensive; often conflict-prone; and require substantial preparation, practice, and trust among team members to ensure a modicum of success [7]. A growing number of studies focusing on the processes and outcomes of trans-disciplinary scientific collaboration suggest that the effectiveness of team initiatives is highly variable, and depends greatly on certain contextual circumstances and collaborative readiness factors [8,9].

### 2.1. Trans-Disciplinary – Definition and Methodology

Before giving a specific example, and a critical assessment, of our involvement in a transdisciplinary research project, it is important to give a concise definition that distinguishes this methodology from others. The first quote has shaped our thinking from the time a former academic colleague, and now medical practitioner, drew our attention to the methodology whilst undertaking a book review. The second quote is the most recently published and suitable definition for our purposes that we could identify:

“Transdisciplinary thinking is primarily a process of assembling and mapping the possible interconnections of disciplinary knowledge about any given…problem until the fullest possible understanding of the problem emerges” [10].

“Transdisciplinarity is an integrative process in which researchers work jointly to develop and use a shared conceptual framework that synthesizes and extends discipline-specific theories, concepts, methods, or all three to create new models and language to address a common research problem” [8].

Both sources identify the differences between the trans-disciplinary approaches and single, multidisciplinary, and inter-disciplinary approaches in convenient tabular form. Furthermore, transcending disciplinary boundaries also requires consideration of different types of integration [11]. “Horizontal” integration is defined as integration across knowledge perspectives, such as disciplines or sectors; “vertical” integration means integration among different types of knowledge users, and may include perspectives from academics, as well as local communities and cultures, and non-government organizations (NGO) staff, for example.

Collaborations by scientists with policy researchers may improve the likelihood of translating research findings into changes in policies, and practices. For instance, multilevel interventions based on ecological models and targeting individuals, social environments, physical environments, *and* policies must be implemented to achieve behavioral change in physical activity in the four domains of active living: recreation, transport, occupation, and household [12]. Research into both environmental and policy influences on physical activity is well underway in many countries: the 2008 Active Living Research Conference theme was “Connecting Active Living Research to Policy Solutions”. Selected journal papers include: principles for improving the translation of research into policy; improving the rigor of research methods; asking policy-relevant questions; presenting country-specific data; and communicating effectively the research findings to policy makers [12].

The main steps of the trans-disciplinary approach [10], which are best illustrated by an example, are:

- problem definition;
- assembling a team of researchers;
- reviewing existing knowledge on the research problem, especially disciplinary and interdisciplinary conceptualizations and explanations;
- designing the research enquiry from research gaps; implementing the research enquiry;
- refining conceptual understandings and synthesizing data sets; and
- recommending the types of interventions (usually with stakeholders) to resolve the problem.

The steps are illustrated briefly by making a critical assessment of a completed trans-disciplinary project into one of the major environmental stressors of transport in major urban areas – that of aircraft noise in neighborhoods surrounding major international airports.

### 2.2. Example of Aircraft Noise and Community Health (Stress)

The context for the research project on the community impacts of aircraft noise was as follows. The Botany Bay Studies Unit at the University of New South Wales, Australia [13], was established as a cross-faculty and cross-university research centre to focus on issues within an area defined broadly as Botany Bay and its hydrological catchments of the Georges River and Cooks River – an area of 960 sq. km., with 14 local government authorities and containing about 40 per cent of Sydney’s population, which is currently around 4.5 million people. Botany Bay is one of Australia’s most important social environments (Aboriginal heritage of settlement and land management, European colonization, industrial heritage and the growth of seaside suburbia) where there is a strong interaction of the natural and social environments. The state government was conducting a sub-regional planning study at the time and there was a cabinet minute allocating $2 million for independent research into problems associated with the region. The former Director of the Botany Bay Studies Unit (Professor John Benzie) and Professor Tony Underwood (Sydney University) were members of the advisory committee in the formulation of the New South Wales Government’s Draft Botany Bay Strategy.

The research problem addressed by the research team was suggested following wide stakeholder consultation. The views of 24 key individuals representing a wide range of stakeholders were sought to formulate research issues. A stakeholder forum, Botany Bay – Moving Forward, was held on 28 February 2004 to move forward from previous studies, to identify research gaps and capabilities of researchers, to formulate a draft set of research priorities. The key questions for participants were “what we don’t know, and how research could contribute to making the study area more sustainable”. In addition, the forum actively sought public submissions on research needs, priorities, and information on relevant studies already conducted. It should be noted that the University provided exemplary support for what eventuated as a trans-disciplinary research study. The Office of the Vice-Chancellor, sponsored the cost of the venue hire – The Scientia Building. Mr Norm Newlin delivered the “Welcome to the Land” address on behalf of the indigenous owners of Botany Bay. The NSW Department of Infrastructure Planning and Natural Resources contributed along with the Botany Bay Studies Unit to the costs of the catering for the workshop. The Sutherland Shire Environment Centre had widely advertised the workshop and this helped ensure important community and NGO participation.

The forum nominated community impacts of aircraft noise as a priority research topic – hardly surprising given that the Sydney International Airport has two parallel runways that project into Botany Bay [14]. The current practice in quantifying aircraft noise retards the resolution of aircraft noise problems as far as the community is concerned because of the misunderstandings surrounding the use of aircraft noise metrics, and an underestimation by the government of public health impacts from long-term exposure to chronic aircraft noise. The sound equivalent energy technique with time-of-day weighting (such as Australian Noise Exposure Forecast and Day-Night Average Sound Level (DNL)) was designed for land-use compatibility advice and regulation around commercial and military airports [15]. However, it has been widely interpreted as a metric correlated with community annoyance. Its application to the study of community reaction to the effects of aircraft take-offs, landings, and over-flights have been criticized [16]. Therefore, new metrics [17,18] and impacts were investigated.

The core team responsible for the design and implementation of the research project came from different undergraduate disciplines: civil engineering; human geography, mathematics and statistics; and mechanical engineering. Such descriptors of a trans-disciplinary research team can be misleading because additional postgraduate qualifications acquired by the team were education; environmental noise, evidence-based medicine; transport engineering; and urban and regional planning. Officers from the Commonwealth Government, through AirServices Australia and the Department of Transport and Regional Development, provided data on aircraft movements and policy advice, respectively. Finally, translators from the South Sydney Area Health Service assisted in the translation of the questionnaire and covering letters into the main languages spoken in the relevant communities that were surveyed. Guest appearances on a local community radio station “Green City” program kept the activities of the Botany Bay Studies Unit in the public mind by explaining the significance of the research and publicizing the survey.

The following steps of the trans-disciplinary process – reviewing existing knowledge on the health impacts of aircraft noise, especially the disciplinary and inter-disciplinary conceptualizations and explanations; designing the research enquiry from research gaps identified; implementing the research project; and refining conceptual understandings and synthesizing data sets – have been reported in the literature [19,20]. One indicator of the quality of the research output is the fact that the paper presented at the 10^th^ Air Transport Research Society World Conference, held in Nagoya, Japan, was one of the seven papers selected for a special journal edition [21].

As for specifying types of interventions to resolve the problem, this is where the project floundered. Stress management techniques were identified for trial in the proposed research proposal based on the cognitive behavioral therapy literature, especially on chronic pain management and on severe asthma [22]. We invited a medical practitioner with international standing in Sahaja Yoga to join the research team. One hypothesis that we hoped to test is that the stress and other health problems caused by aircraft noise can be ameliorated by non-chemical complementary medicine stress management interventions [23]. This hypothesis is based on understanding the neuroscience of the brain and its response to noise as an environmental stressor. We planned to implement an intervention with aircraft noise affected residents of Sydney (a parallel research proposal would examine stress in the workplace and evaluate SMI). However, the research grant applications were not funded, and, as is common in the case of researchers assessed by their peers as “internationally competitive”, other projects were pursued, the trans-disciplinary research impetus on aircraft noise and stress was lost and the core team has subsequently disbanded – by moving to other universities, or retiring.

Elsewhere, we have argued for the case for the application of trans-disciplinary research into cities, transport, quality of life and environmental health [21,24,25]. Therefore, in the next section we confirm the research relevance of these specific domains and attempt to identify trans-disciplinary research on urbanization transport and public health.

### 2.3. Trans-Disciplinary Research into Transport and Health

Given the importance of the transport sector of the economy, the contribution of the road sector in particular to greenhouse gas emissions [26] and the direct and indirect impacts of transport and urbanization on human health we might expect there to be reports published studies organized by the trans-disciplinary approach. Three search methods were used. First, the International Association for Ecology and Health focuses on research and practice that integrates human, wildlife, and ecosystem health and its understanding, draws on integrative and cross-disciplinary approaches involving both the ecological and health sciences. An editorial overview suggests that the common, overarching purpose of ecology, health, and sustainability research domains is to better understand the connections between nature, society, and health, and how drivers of social and ecosystem change will influence human health and well-being, where a trans-disciplinary approach is advocated [27]. Socio-economic change, public health initiatives, and gains in medical care have continued to improve basic health indices in recent decades, but economic development (which includes urbanization and transport infrastructure development) can impair public health if environmental and social considerations are neglected.

Secondly, the research activities of the World Conference on Transport and Research Society (WCTRS) – the pre-eminent society for all disciplines associated with transport covering academia, professional practice and policy makers – were examined to see if the emerging link between transport and health had been identified in any of the Special Interest Groups [28]. Finally, we used key words to locate trans-disciplinary studies of urbanization, transport and health, restricting the computer-based search using Medline but found few such over-arching studies reported in the literature, and none from Australia.

Research teams examining the nexus between urbanization, transport and health appear not to have chosen the *EcoHealth Journal* [29] for placement of their research output. Of the 295 articles published between March 2004 and March 2009 only three within the domain of our interest could be located. Only one study aimed to validate a new index of the bio-psycho-social cost of ecosystem disturbance [30] could be assessed with confidence as following the trans-disciplinary model because the innovative environmental stress scale successfully measured and validated the concept of “solastalgia” – the sense of distress people experience when valued environments are negatively transformed [31]. In a descriptive narrative, the ‘cases’, the high disturbance group, experienced greater exposure to dust, landscape changes, vibrations, loss of flora and fauna, and building damage, as well as greater fear of asthma and other physical illnesses due to local pollution than the ‘control’ group. Although this study was informed by fieldwork in the open-cut mining area of Australia’s Upper Hunter Valley, it could be adapted as a general tool to appraise the distress arising from people’s lived experience of the desolation of their home and environment – for example, those households in proximity to the construction of major transport infrastructure.

The most relevant inter-disciplinary article on transport [32] by sociologists and psychologists, analyzed in-depth interviews about the meaning and significance of social and recreational travel (a proxy for social capital) in Auckland, New Zealand, for a diverse group of Maori (indigenous people of Aotearoa/New Zealand), Samoan (originating from the Pacific Island of Samoa), and Pakeha (a Maori term commonly used to describe New Zealanders of European ancestry). The findings highlight the benefits of social and recreational travel both for maintaining social and family relationships and for general health and well-being, including the opportunity to participate in physical activity and in other activities that help reduce stress.

A general, comparative, article on urbanization, *inter alia*, describes air quality trends in the “sister” cities of Wuhan, China, and Pittsburgh, USA and offers a brief qualitative description of changes to the built environment – albeit with no assessment of health impacts. The only explicit reference to transport is that in Wuhan travelers have switched over time from walking and cycling to using public transport [33].

A scan of the nearly 1,000 paper titles at the 11^th^ World Conference on Transport Research, hosted by the University of California Berkeley [34] also confirmed that trans-disciplinary research studies were equally rare [35,36]. Researchers and practitioners of environmental health are poorly represented reflecting the fact there are more appropriate societies for their disciplinary interests. This is somewhat surprising given that transport activities impact on health, both negatively and positively; and transport policies are now a key determinant of health [37]. As would be expected, there were specialized sessions in the conference program dealing with transport safety – especially road safety – and security.

**Figure 1:**
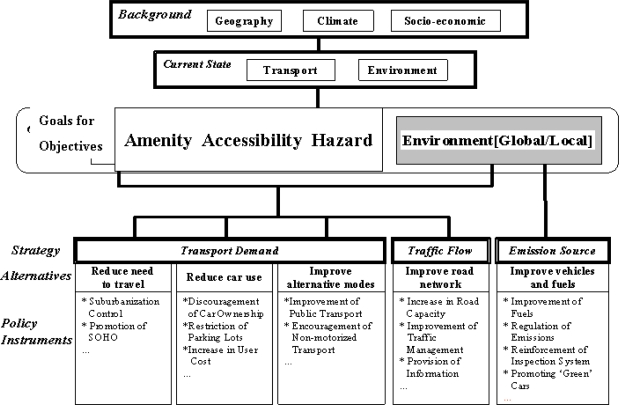
Goals, Strategies and Policy Instruments for Integrated Land Use and Transport Solutions to Environmental Problems. Source: [30].

That there were so few papers relating to urbanization, transport and environmental and health impacts was somewhat surprising given the Society’s earlier initiative with a multi-disciplinary, multicountry study of transport and the environment, with the Editors achieving after much debate and argument a common conceptual understanding. Figure 1 illustrates a similar diagrammatic representation of goals, strategies and policy instruments for integrated land use and transport solutions to environmental problems in Japanese cities [24]. The World Conference on Transport Research Society (WCTRS) and the Institute of Transport Policy Studies, Tokyo used a similar conceptual model to guide research collaboration on urban transport and the environment [38].

## 3. Conceptual Representation

All models are designed for a specific purpose. Our conceptual representation of the social and environmental (primarily human settlement and transport driven) determinants of health and wellbeing through the life-span of individuals is no exception. Given as a staring point the transdisciplinary focus referred to in Section 2 the model is designed to bring together some of the enabling scientific components from a range of disciplines as they might bring to bear on the problems, analyses and solutions in this conceptualization. Essentially, the purpose of this model is a scoping one that will allow us later in Section 4 to classify the literature more systematically, and to help identify gaps in knowledge, especially as this might relate to one of the key drivers of environmental change – climate change (Section 4) and the particular risks likely to affect human settlements [39–41]. The components of this model are best explained using a hypothetical region.

In the region of our conceptualization - delimited by its geographical boundary (an assumption later relaxed) – we could consider its sustainability in terms of seven major themes: Atmosphere, Land, Inland Waters, Coasts and Oceans, Biodiversity, Human Settlements, Natural and Cultural Heritage (common categories for State of the Environment reporting). But we are interested in health and sustainability [1] so the prime object are the people, their exposure to environmental stressors and the cumulative effects of exposure to these stressors over their lifetime. The focus on people is because “population health and well-being is the ‘bottom line’ of sustainability” [42]. The purpose and objectives of trans-disciplinary research when applying the model into the specific health impacts from human settlements and transport are to provide accurate, up-to-date and accessible information on the state of conditions, trends and pressures on people’s health (with full cognizance of the ageing process), and to articulate responses by the way of solutions and reporting to key stakeholders who are responsible for the governance of the region.

The model proposed to do this is a perfectly general one that reflects the causal relationship between human activities, the pressures they place on the environment, the feedback those activities have on people through environmental pressures, and the actions that are taken in response to these pressures. It is straightforward to see that it is a standard “state of the environment” approach where the DPSIR Indicator Framework [43] – “Driving” forces of environmental change, “Pressures” on the environment, the “State” of the environment; “Impacts” on population, and the “Response” of the society – can be readily adapted. The pressures come from the way human settlement patterns and their transport systems are deployed and utilized by people going about their daily activities, and the associated impacts these have on the health of the population. As our bodies age, our ability to defend against environmental pressures diminishes, and exposures can accelerate the aging process and trigger, or exacerbate, disease. As stated by Hood [44] “decreased efficiency in the blood-brain barrier and the cardiovascular, pulmonary, immune, musculoskeletal, hepatic, renal, and gastrointestinal systems can alter response to environmental agents, leading to heightened susceptibility to the toxic effects of air pollution, pesticides, and other exogenous threats to health.”

At this point of the exposition, it is important to recognize that, across the world, there are widely varying climate zones and socio-economic variations and human activity patterns that will influence the local details of the environmental stressors, the relative importance attached to them given the values of each society and the responses by the respective public and private sectors of the economy depending on the governance and political economy of each region. The application of the proposed model would capture these contextual differences in the specific research problem statement. For example, and to take two extreme situations, the challenges of sea-level rise as a driving force of environmental change will impact on large populations living in the deltas of many great rivers of the world [45], whereas in arid countries, such as Australia, water security becomes an issue with projected increases on annual mean temperature [46].

To validate the proposed model, research into the above factors in any real region should conducted within a trans-disciplinary framework with team science (see, Section 2 above) that also considers society’s response to the environmental pressures and impacts on this life-span aging process by assessing interventions (using economic, social and environmental evaluation methodologies) in an over-arching sustainability framework of alternative scenarios, policies and programs [47,48]. Translation of findings into practical outcomes with key stakeholders is a key goal in a comprehensive evaluation of the model.

The scientific rationale of the model derives from four main concepts. The first is founded on the literature on quality of life especially that of a multi-dimensional conceptualization with five categories of concepts and domains of health - opportunity; disadvantage; health perceptions; functional status; impairment; duration of life and death (Table 1). This represents the way we think about the health and well-being of the individuals studied and how we might evaluate the outcomes of any intervention. Health outcome can only be measured within the constraints of the person’s environment and their perception of their health [49].

**Figure 2:**
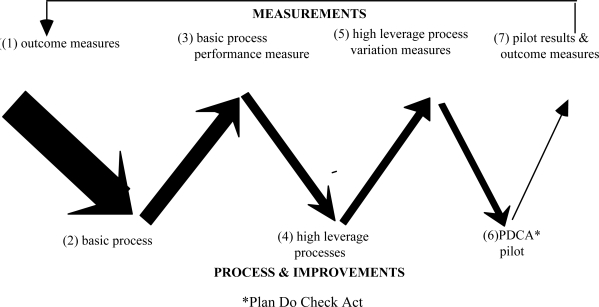
Diagrammatic Model of the ‘Serial V’ Concept in Continuous Quality Improvement.

Also, the ‘Serial V’ concept, that integrates the health outcome measurement, process improvement and continual improvement (Figure 2) can form part of the evaluation methodology. The outcome measures can relate to: mortality and morbidity rates; physical, mental and social functioning; satisfaction; quality assurance monitors; and cost/resource usage [50]. The basic process measures might be speed, accuracy, appropriateness and efficiency. The high leverage processes are more difficult to identify and require both professional and local knowledge and analyses of cause-and-effect.

The second concept, which is derived from work by Swedish geographers led by Professor Torsten Hagerstrand [51] on “time-space” geography, allows for an accounting of peoples’ geographical location in the space identified as the region, and the human activities that they undertake in that region. Theoretically, this accounting should take place with longitudinal studies from “cradle to grave” to account for the aging process but the costs and difficulties of such a survey preclude this full accounting [52]. This human activity approach – “the things people do in time and space” [53] with its associated survey methodology [54] – leads to the analysis of the precise location, activity, and time-duration for each individual (with its unique identifier of demographic and socio-economic condition as identified in the first component of the model) and their space-time exposures to the environmental stressors – as conceptualized by Hagerstrand [55,56]. It is especially important to note that survey data from the human activity approach will reveal all travel – which includes the origin and destination of the journey, the route taken, the transport mode taken (walking, cycling, public transport and private transport) and the duration of those journeys. The time spent at various origin and destination locations in the region, including, importantly, in the home, will be an important factor in calculating environmental exposure to stressors such as pollution from the transport systems. Also, the duration of time spent in walking and cycling (plus other physical activities such as sport) will give quantitative information on whether that person is leading a “healthy” or sedentary life-style at that particular stage of the persons aging process.

The third component of the model is the associated fundamental science of the stressors under investigation in the region and their impacts on the population – of which a large body of literature may be accessed. Analysis is based on the physical properties of the various stressors (exogenous threats) that have bearing on the health of the population, including the precise amplitude and location by time of day of these stressors across the region. For illustrative reasons only, we select vehicle emissions and transport noise. The combustion of gasoline and diesel fuels in an internal combustion engines results in tailpipe exhaust emissions that mix in the atmosphere and disperse according to specific meteorological conditions that constitute greenhouse gases, suspended particulate matter and contribute to the formation of smog. These fundamentals are complicated because the rate of emissions will depend on a variety of factors including vehicle type and age, standard of maintenance and the traffic flow conditions influencing driving behavior and speeds. These complex interactions are well-described in the body of knowledge encompassed in traffic engineering [57].

Sound pressure waves generated by transport vehicles, and their interaction with the road pavement surface (traffic), rail (rail-based freight and public transport) and the air (jet aircraft) propagate from a multitude of sources with the region according to well understood principles of physics. However, it is the acoustical energy of differing wavelengths, again tempered a little by prevailing meteorological conditions that the human ear receives and translates this into the brain as un-wanted sound or “noise” (primarily a psychological reaction that is somewhat subject specific). Psychologists and acoustical engineers have developed a thorough understanding of exposure to environmental noise (dose-response relationships).

The fourth component of the conceptual model is to exploit both the rapid developments on computing technologies for the analyzes and linking of large data bases and the power of geographical information systems (GIS) and the software commercially available to map layers of interest at specific locations across the region, or in a sub-set study area. Visualization using GIS allows layers associated with the seven main themes of environment and heritage to be mapped, and the place and time-dependent nature of the stressors to be superimposed (primarily line sources in the case of most transport infrastructure but three-dimensional plots of flight paths for airports and aircraft noise). The integration of land information systems, mathematical models of travel demand and of road traffic noise and the exposure of neighboring properties to noise impacts have been achieved in a GIS framework [58–60]. It is the time-varying location of people going about their daily activities in the region and the different duration of those activities in geographical space that, when mapped into locational patterns of the stressors that will enable exposure to be estimated. Of course, GIS has other important uses in validating the model such as the mapping of land use locations (for studies of accessibility, social capital and well-being) and of traffic accident locations.

A final observation on the model is that it can be further revised and refined following the formulation of hypotheses and their testing in real regions of cities and their inter-connecting hinterlands. Suitable analytical tools can be drawn from evidence-based medicine, epidemiology and statistics, systems and risk analysis (widely used in practice by environmental and transport engineers). Risk assessment procedures in the US regulatory agencies came from the US National Academy of Sciences. The formalization of risk assessment and risk management found its way into international organizations, federal agencies and business during the 1980s. More recently, global climate change risk is seen from both broad concepts of mitigation and adaptation. This latter point is a reminder that the hypothetical region that we have outlined has a boundary that is not immutable and so is subject to trans-border events - both of a physical (climate) and a social (human migration) dimension.

## 4. Literature Review

### 4.1. Literature on Human Settlements, Density and Heath

There is a long history of writing on the environmental problems of the 19^th^ century industrial city, and the contemporary city, such as the architectural guide attempts to define a theory and language for constructing spaces that allow for optimal human happiness and well-being [61] and the effects of traffic on the urban environment [62]. The influence of built-form factors on health (and well-being) is now well established from evidence cited in an extensive literature [63–71]. There is a body of literature that links urbanization and urban layout with obesity, especially the influence of low-density suburban developments that are car dependent and encourage sedentary life styles, including chauffeuring children around in motor vehicles. In 1996, the US Surgeon General’s report on physical activity and health established the multiple health benefits of physical activity and contended that thirty minutes of moderate physical activity several days per week could help to prevent a range of diseases [72]. The Robert Wood Johnson Foundation’s multi million-dollar Active Living portfolio aimed to increase understanding of the policy and other forces that shape the built environment and to alter them to support the formation of environments that are more amenable to physical activity. Active Living by Design funded twenty-five communities [73]. The revised mission from 2007 to 2012 is to stimulate and support research on environments and policies that influence physical activity to inform effective childhood obesity prevention strategies, particularly in low-income and racial/ethnic communities at highest risk. Active Living Research (ALR) examined and measured the design features of communities and charted their connections to levels of physical activity, aiming to accumulate evidence on how the built environments of a range of communities shaped physical activity. However, built environment characteristics near home did not consistently predict walking for exercise in a healthy population in western Washington State, USA. Further, there was little evidence of neighborhood-level variation in walking for exercise, despite neighborhood-level variation in the built environment [74].

The policy changes to which the active-living partners aspired rarely came quickly or without conflict [75] as documented in the special edition of the *Journal of Health Politics, Policy and Law* [76]. Some of the suggestions that focus on fixing the problem involve transport at their core: collect travel and activity data from people before and after rail lines open or after cycling and pedestrian improvements have been made to analyze the link between investment interventions and travel and health benefit outcomes; require that health criteria be at least considered within cost - benefit analyses across transport modes to be achieved by providing incentives for government bureaucrats to evaluate the health impacts of alternative transport investment; and address transport-related impacts on sedentary behavior through increased funding for public transport, walking facilities and bike paths. Doctors for the Environment Australia [77] are also advocating government spending on public transport through a national initiative to all Members, Senators and Ministers in federal parliament seeking their recognition that public transport is a climate change and a health issue.

The economic dimensions of the problem of transport policy that has favored developers in suburban regions and promoted private vehicle usage and road building programs in the USA are becoming clearer. Recently, a submission by the American Public Health Association [78] at a workshop on the Hidden Health Costs of US Transportation Policy estimated the annual costs to be: traffic injuries and fatalities at about $200 billion; obesity/overweight societal cost at about $117 billion and the cost of inactivity at about $76 billion; and air quality from $40 to $64 billion. International comparative studies on such costs should be updated regularly as additional data becomes available.

### 4.2. Literature on Transport Impacts on Health

**Figure 3:**
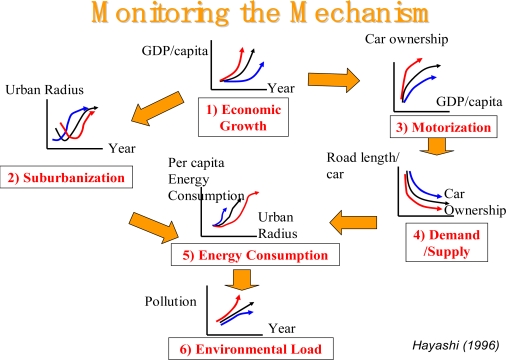
The Processes of Urban Development Explained with Key Mechanisms. Source: [82]

From the earliest days of the research that has now blossomed into the World Conference on Transport Research Conferences (see Section 3), urban land use and transport have been analyzed together [79], and their interactions quantified as a key process [80,81]. The dynamics of the driving forces in the DPSIR Indicator Framework for human settlements and transport is now firmly established [82]. The key mechanisms in this driving force are illustrated graphically in Figure 3 as a systems flow diagram. It is worth observing that mathematical models of demand and supply (mechanism 4) have been exhaustively refined and verified by researchers over a long period of time. One of its leading researchers – Professor Daniel McFadden, a Nobel Laureate on consumer choice behavior – gave the keynote address at the most recent World Conference on Transport Research held at the University of California Berkeley in June, 2007.

Monitoring macroscopic changes in transport and land use is a useful starting point, although transport planners would analyze detailed traffic movements and land-use change at the fine geographical detail of micro zones and consider transport pollutants at the more macro, urban level [83]. Figure 3 shows a general set of simple indicators for monitoring change are shown [84], where the blue lines are the early phases and the red lines the latest phase of urban development. In simple outline, the processes of development are driven by economic growth (income per capita) which in turn leads to increased motorization (car ownership); high road congestion levels (road length per registered car), which introduces additional air pollution. Economic growth is a driver of more residential space per person; an increasingly sprawled city (urban radius); poor accessibility with increasing spatial separation and longer journeys (total trip length). In a sprawled, car dependent city total energy consumption (and associated tail-pipe emissions) plus the carbon burden from automobile manufacture for transport is high and this is exacerbated by road congestion. That such a pattern of urban development is unsustainable has been clearly documented by numerous authors, one of who[84] is widely cited in the mainstream transport literature.

The main stressors from urban transport systems (only pollution is shown in Figure 3) are: accidents involving road vehicles, cyclists and pedestrians; transport noise and vehicle emissions (and ambient air quality). First, we will mention only in passing traffic accidents because its causes (human factors, vehicle and road environment) and countermeasures are extensively documented in specialist journals. For example, the *Journal of Accident Analysis & Prevention* [85] provides wide coverage of the general areas relating to accidental injury and damage, including the pre-injury and immediate post-injury phases. Published papers deal with medical, legal, economic, educational, behavioral, theoretical or empirical aspects of transportation accidents, as well as with the accidents themselves.

Secondly, the analysis and prediction of aircraft, road and rail traffic noise is well established health effects of transport noise are well documented [86–89]. There is a large amount of evidence that negative emotional states are acutely associated with cardiovascular pathophysiology [90–92]. The evidence about the world-wide distribution and cause of the aircraft noise problem in suburbs surrounding airports is also compelling [93]. Annoyance from aircraft noise, is well documented in the literature [94,95], but stress and hypertension has only been identified in more recent years [96–99]. In our own contribution to this topic a self-reported questionnaire using the validated instrument SF-36 measured health quality of life, prevalence of hypertension, chronic noise stress, noise sensitivity, noise annoyance, confounding factors, and demographic characteristics [21]. Aircraft noise is one of the best illustrations of environmental stressors that are a major component of sustainable health in cities. For example, research on aircraft noise was of sufficient social importance to be included in *The Sydney Morning Herald, Sydney Magazine* (Issue #60 of April, 2008, p. 58) [100].

Thirdly, disentangling the impacts of transport pollutants from other pollutants in the atmosphere is more difficult. Determining the risk posed by environmental pollution to public health [101] requires knowledge of five fundamental components: the source of pollutants; the transport of pollutants from sources to humans; the exposure of humans to pollutants; the dose response for those exposed. There is considerable variation between subjects, for example, there will be a large difference between those exposed and not exposed to environmental tobacco smoke [102]. Vehicle emissions are an important source of a number of potentially hazardous air pollutants, including particulate matter, nitrogen dioxide, carbon monoxide, and several air toxics. Although questions remain about some of the chemical and biological processes at play, including whether there are synergistic effects of combined pollutants in the atmosphere [103], there is strong evidence that many of the pollutants emitted by motor vehicles pose serious health risks, and that those risks are elevated for individuals, especially sensitive individuals, living in close proximity to a high-traffic road.

The negative health effects of many of these pollutants have been well documented [104]:

- “Exposure to fine and coarse PM in ambient air has been associated with a short-term increase in mortality and morbidity from cardiovascular and respiratory diseases.
- Studies have found long-term average mortality rates 17% – 26% higher than expected in communities with high levels of fine particulate matter.
- Diesel exhaust and nitrogen dioxide have been tied to increased asthma symptoms and response to allergens.
- Exposure to diesel exhaust has also been associated with increased rates of lung cancer and mortality and morbidity.
- Air toxics can cause negative health effects including cancer and respiratory, neurological, reproductive, and developmental effects.”

The health risks posed by particulate matter (PM) in ambient air are a cause for concern (for a recent overview of research in the USA, see [105]). Fine and ultra-fine particles, because of their small size and/or their chemical composition tend to be more hazardous to human health than coarse particles. Results indicate an elevated mortality risk from short-term exposure to ultra-fine particulates [106]. PM from motor vehicles in particular has been shown by several studies to be more toxic than PM from other sources. Ambient concentrations of particulate matter have been consistently associated with daily mortality [107–109]. Associations between ambient concentrations of nitrogen dioxide (NO_2_), carbon monoxide (CO) and daily mortality have been observed [110,111], however, the causality of the NO_2_ effects is currently being debated [112].

Exposure to traffic-related pollution is a complex subject. Residential traffic is associated with both current symptoms and prevalence of diagnosis of asthma and chronic bronchitis, among adults in southern Sweden. Traffic has not only short-term but also long-term effects on adult chronic respiratory disease, even in a region with low overall levels of traffic pollution such as Southern Sweden [113]. Traffic activity increases the level of air pollution, with locations near heavy traffic having significantly higher particulate levels than those locations near lighter traffic [114–118]. A study of cyclists in Mol, Flanders [119] found relatively higher ultra fine particle concentration exposure during morning office hours and moderate ultra fine particle levels during afternoon. The major sources of ultra fine particles and PM_10_ were identified from vehicular emission and construction activities, respectively. In fact, air-quality models based on traffic patterns account for up to 50–73% of the variability in average annual levels of fine particulate matter [115,118,120]. Although ambient air quality can vary within a city, generally, people living near high-traffic roads have the highest levels of exposure. Wind speed and direction and building heights can also impact pollutant distribution, adjacent to the roadway and across a metropolitan area [121].

Both PM and ozone can be transported long distances, impacting ambient air quality over a wide area and limiting the effectiveness of local pollution control efforts. Ground-level ozone and PM_2.5_ have been linked to negative health impacts ranging from minor respiratory problems to cardiovascular disease, hospitalizations and premature death. For example, based on data from eight Canadian cities, Health Canada has estimated that 5,900 premature deaths each year in these cities are attributable to air pollution [122]. In the city of Sao Paulo, Brazil, logistic regression revealed a gradient of increasing risk of an early neonatal death with higher exposure to traffic-related air pollution [123].

A large body of research suggests that the typical extent of elevated exposure to PM_2.5_ and nitrogen dioxide is roughly 100 to 500 meters from a major road [124–127]. Studies examining the impact of living near major roadways and the consequent long-term exposure to traffic-related air pollutants have shown a variety of health risks, including:

- significant increase in the risk of death from cardiopulmonary causes
- significant increase in asthma prevalence in children
- impacts on lung development in children
- increased cardiac arrhythmias

Sensitive individuals, who are more likely to experience these effects, include the elderly, those with influenza [124], in asthmatic children [125] and in diabetic subjects [126], and in younger children (in a nationwide US study [127], increased respiratory allergy/hay fever was associated with increased summer ozone levels and increased fine particulate matter). Further research that classifies and analyzes the population by age and socio-economic characteristics and their exposure to pollutants when going about their daily activities in places other than the home is desirable.

There is a strand of research that looks at the stress involved primarily from driving private motor vehicles (the trauma of the road toll could be included as a negative impact of using transport) and driving trucks as an occupation [128–132]. The extent of metropolitan congestion is increasing in Australia: for example, motorists in Melbourne are spending an extra day a year behind the wheel. A typical driving commuter spends almost two weeks - about 336 hours - a year going to and from work. In 1999, the same driver would spend only 12 days and 17 hours in the car every year for the same trip [133]. Traffic speeds for commuters to the Sydney CBD who use the M2 toll road, the Lane Cove Tunnel and the Gore Hill Freeway fell to just 31 kilometers an hour in 2007–08, down from 38 kilometers per hour a year ago [134]. In a study of the US National Household Travel Survey [135], higher commuting time (more than 20 minutes) was significantly associated with no socially-oriented trips (as a proxy for social capital)

Finally, by way of an observation on the solutions and countermeasures to the environmental stressors and their impacts: these are usually well covered in governments state of the environment reporting for the national regional and, sometimes, local levels. For example, searches can be made of the various Australian state government policies and programs in the various state of the environment reports [136]. A systematic review of the evidence on the most effective ways of improving population health through transport interventions [137] is now dated and requires updating. An international comparative analysis of societal responses within the DPSIR Indicator Framework, much like the comparative analysis of cities, as summarized in Figure 1 of this paper, would give a lead as to what policies and programs have been successful in a range of urban situations.

### 4.3. Literature on Visualization and GIS

Visualization of those environmental stressors (and health impacts) from the urbanization process outlined in Figure 3 is highly desirable, especially when communicating scientific information to stakeholders and wider publics. Geographic information systems (GIS) provide ideal platforms for the convergence of locating environmental stressors and public health information the natural and manmade environment. They are highly suitable for analyzing epidemiological data, revealing trends and interrelationships that would be more difficult to discover in tabular format. Moreover, GIS allows policy makers to easily visualize problems in relation to existing health and social services and the urban environment, and so more effectively target resources. The World Health Organisation (WHO) has a public health and GIS mapping program - but this is only at the global or national scale and not at the spatial resolution of the city. The logical extension of this visualization challenge of GIS is to apply it to urban areas, human activity patterns and environmental stressors.

As a starting point in the development of such a system there are books on GIS and public health [138], public health information visualization technology [139] and a range of recent University initiatives linking geography, web-based spatial analysis (GIS) and epidemiology (for example, McMaster University, Canada, University of Iowa, Improving Public Health Through Geographical Information Systems An Instructional Guide to Major Concepts and Their Implementation Web Version 1.0 December, 1997 [140,141] and disaster management, emergency planning and responses to terrorist attacks [142–144].

A number of geographic perspectives on health and environment could create useful connections between geography and public health, via social epidemiology [145,146] – for example, the dust map, or a graphic presentation of particulate concentration, where the particulate concentration ranges are projected on a street plan or aerial photograph [119]. Measured particulate concentrations are coupled with the GPS positions and then projected on the entire transport route (along which individuals pass and are exposed to the pollutant).

In order to properly plan, manage and monitor any public health program, it is vital that up-to-date, relevant information is available to decision-makers at all levels of the public health system. As every environmental stressor requires a different response and policy decision, information must be available that reflects a realistic assessment of the situation at the local level. Geo-coding accuracy has been established for environmental exposures and health [146]. This must be done with best available data and taking into consideration, demographics, availability of, and accessibility to, existing health and social services as well as other geographic and environmental features, including climate change impacts. Improved information systems, which are an integral part of outcomes assessment and a continuous quality improvement approach advocated in Section 2, will result in more effective decision making. If health information systems are to make a practical contribution to the health system then there is a need to measure the output concisely [147]. These comments are of particular relevance to integrated solutions for the sustainability of cities and regions include public health informatics and outcomes assessment. A stakeholder survey of 522 leaders and professionals in the 25 largest cities of the world found that health care is a major infrastructure challenge, and furthermore noted that IT in health care has a major role to play, supporting both treatment and administration [148].

### 4.4. Future Issues – Climate Change

Within most metropolitan regions of the world there is evidence that environmental stressors are increasing and health impacts will be exacerbated by climate change in the 21st century [149]. Public health specialists have raised the potential impact of climate change on health and there are several research topics emerging. Changes in temperature, humidity, rainfall, and sea level rise could all affect the incidence of infectious diseases, and this is the most common topic [150,151]:

- Association between heavy rainfall and Ross River virus disease.
- Both insects and insect-borne diseases (including malaria and dengue fever) have been experienced at increasingly higher altitudes in Africa, Asia and Latin America.
- Heavy rainfall may cause outbreaks of cryptosporidiosis which causes severe diarrheic diseases in children and can cause death in immuno-compromised individuals.
- An increase of the temperature can activate the blooms and vibrios (cholera) in fishes.
- The emergence of Hantavirus pulmonary syndrome may be linked to heavy rainfall resulting in growth in rodent populations and subsequent disease transmission.
- Extreme flooding or hurricanes can lead to outbreaks of leptospirosis and by their violent nature, natural disasters like storms, floods, cyclones, have the potential to cause morbidity, mortality, and property loss [152].

A comprehensive and recent review of climate change and human health research needs in Australia may be found in a late 2008 report [153].

Heat waves are expected to increase in frequency, intensity and duration this century [154,155] and the urban heat island effect is likely to cause additional problems in cities. Mortality from heat waves is related to cardiovascular, cerebrovascular, and respiratory disease and is concentrated in elderly persons and individuals with pre-existing illness, and deaths have been examined in Europe and the USA [156–158]. A study of the 2006 Californian heat wave [159] analyzed county-level hospitalizations and emergency department visits for all causes and for cause groups. During the heat wave (July 15-August 1, 2006), excess morbidity and rate ratios were calculated and compared to July 8–14 and August 12–22, 2006. Emergency department visits for heat-related causes were found to increase, especially in the central part of the state which includes San Francisco. Children (ages 0 – 4 years) and the elderly (ages ≥ 65 years) were found to be at greatest risk.

Increases in mean temperatures and more sunny days will intensify another set of problems. Damage to the earth’s stratospheric ozone layer will lead to an increase of solar ultraviolet radiation reaching the Earth’s surface, possibly increasing the incidence of skin cancers [160]. An increase of the global temperature causes earlier pollen seasons and the increase of pollen season duration [161] and the pollen produced may be more allergenic [162]. Quantity and seasonality of pollen are likely to be impacted by both climate-forcing of phenology and direct effects on pollen production - trees in the spring, grasses in the summer; ragweed in the autumn [161]. One of the most common plant-induced health effects relates to aerobiology, including sneezing, inflammation of nasal and conjunctival membranes, and wheezing – in Japan, the cedar tree pollen can be “the enemy of some people”. In addition to increased pollen exposure, there are other consequences of increased fossil fuel burning which may be synergistic; for example, diesel particles help deliver aeroallergens deep into airways and irritate immune cells [162].

Increased temperatures increase the risk of natural forest fires (although some are deliberately or carelessly started by humans) in dry climate zones (a seminar on this topic presented by Professor David Karoly was held in Melbourne, Australia, on 27 March, 2009 [163]). The recent bush fires in Victoria, Australia that started in successive days of hot temperatures ranging in the mid-40°C, were a brutal reminder of the power of Nature. The Deputy Prime Minister of Australia, Ms Julia Gillard, moved a condolence motion during a two-hour sitting of Parliament: “The 7th of February, 2009, will now be remembered as one of the darkest days in Australia’s peacetime history.” [164]. Fires burned 413,000 hectares, destroyed 1,834 homes and more than 400 rental properties in 78 affected communities of regional Victoria. As of 5 March, 2009, the official death toll was 210, but that could rise as police continue their search for more bodies; because of the nature and intensity of the fire some victims may never be known. By early March there were still 1,200 kilometers of containment lines surrounding four burning fires, which fortunately held under the “worst conditions” of temperatures and high, gusting winds on 3 March [164].

Such problems of climate change as a driver of urban policy has been recognized in Australia. The National Climate Change Adaptation Framework (the Framework) was endorsed by the Council of Australian Governments (COAG) in April 2007 as the basis for government action on adaptation over five to seven years, and up to $50 million will be invested in priority research for key sectors as identified in National Adaptation Research Plans (NARP). The main purpose of the NARP for human health is to articulate a research agenda for the next 5–7 years through which to acquire fuller understanding of the health risks from climate change in Australia, and how to reduce those risks via planned adaptive interventions [165]. These research projects are now underway in Australia and the next section speculates on some research gaps from a similar, Australian perspective.

### 4.5. Classification of the Knowledge Base and Research Gaps [166]

First, we will make a brief observation on the state of “team science” in Australia to complement Section 2. Then, based on components of the conceptual representation in Section 3, we will now classify in Tables 2 and 3 the common health impacts from urbanization and transport which have been identified in Sections 4.1 and 4.2. The schema adopted here is the geographical location of people when they are affected by environmental stressors and the geographical scale of that environmental stressor (exposure). Suggestions on obvious gaps in the literature with particular reference to the Australia context are offered. This will help suggest research that will bridge the gap between the traditional, individual-level health care approach and population-based health care [167] and provide also a closer link between research and research-led teaching in universities.

Searches of databases including Medline using keywords such as “public health, urbanization, transport and Australia” were found not to be effective, and, in fact, showed zero citations. However, we are aware of key research centers in Australia that are making contributions relevant to the themes of this paper including a transport component. At the University of Sydney, the Centre for Physical Activity and Health, School of Public Health had conducted research in multidisciplinary research teams on how “walkable” local neighborhoods are. Similarly, The University of Western Australia Centre for Built Environment and Health [168] has an on-going research program focused on examining the impact of the urban environment on health indicators and disease outcomes in children, adults and older adults. It is a fertile field for research in the Australian context given the current state of the science in the USA where a substantial amount of research has been conducted [169] and that given the first comprehensive examination of built-environment measures has only recently been published which demonstrates, despite the considerable progress over the past decade, there still is a need to improve the technical quality of measures, understand the relevance to various population groups, and understand the utility of measures for science and public health [170] and shape a research agenda [171].

The Australian National University National Centre for Epidemiology and Population Health [172] is taking a life-course approach to the study of health and wellbeing, including issues such as: “the prevalence and incidence of health problems varying with age or stage of life; the extent to which the health status and wellbeing of individuals changes over time; the risk factors over the life-course that combine to influence health and wellbeing, through cumulative or synergistic effects; and the combination of environmental influences combined with personal vulnerability (including genetic predisposition) in determining health and wellbeing”.

There is a well-established international public health literature that represents the way in which a variety of influences, including the social environment and the physical environment factors identified in Figure 3 interact to affect individual health and well being but we will cite one important Australian source [173]. There are also examples in Australia of the non-health sectors (including housing and public planning) which may have a role in working with the health sector [174], but these collaborations must be strengthened and solutions considered as an integral part of a trans-disciplinary team. Furthermore, it should be noted that there has been a change in approach to environmental studies within public health that emphasizes the close inter-relationship between human activity, industry, the physical environment, environmental stressors and human disease, ill health and mortality [175].

Selected examples from Australian cities will help illustrate a few of these inter-relationships (Table 2). The main drivers of the problems of Australian cities are probably the develop-led, urbanization process (and consumer preference for the “quarter acre block” of house and garden that has led to low density, sprawling, suburbs, and material affluence with high levels of motorization where accessibility to health (and other) facilities is a key issue, especially for those without an car [176]. Some of the management, mitigation and adaptation solutions are summarized in the right-hand column of Table 2. As with practical experience of urban transport policy [177], integrated solutions across sectors are essential and research can contribute to helping over barriers to implementation and the simulation of all costs and benefits associated with such a coordinated set of policy options. For instance, integrated land-use and transport planning aims to reduce dependence on cars by improving access to public transport, walking and cycling; providing facilities nearby so people travel shorter distances; and encouraging multi-purpose trips, which reduces the total number of trips. The New South Wales Government’s responses to these issues are summarized in its State of the Environment Report [178].

That human health impacts (that are both positive and negative) should be accounted for in the planning, development and management of urban environments given the stressors and impacts identified in this review. Integrated solutions across urban development, transport and health sectors are required. In the Special Edition of the *New South Wales Public Health Bulletin* on ‘Cities, Sustainability and Health’ an example of this approach is proposed with a ten-point checklist for the planning and development of healthy and sustainable communities has been proposed [179]. Developing these sector inter-connections a bit further, an initial research project could be to classify evidence on public health and urban form with particular reference to the location and time spent in high environmental stress areas (exposure) by taking into account individual life histories, where possible, and to teasing out built form effects from socioeconomic confounders, including the role of the law in supporting urban dysfunction [180]. All events that impact on the health and well being of the urban population, whether they have been a result of the built form, in general, or motor vehicles or jet aircraft, in particular, exhibit a geographical pattern of incidence. It is the appropriate management of such factors at the correct spatial level of resolution that presents an action-based research challenge to help contribute to more sustainable cities.

Innovation is necessary to achieve socially-sustainable solutions. Partial solutions generated by traditionally distinct professional disciplines are unlikely to result in real innovation, as argued throughout this review paper, and by others. For example, some of the research challenges for urban researchers from a social–ecological perspective are [181]:

*“The spatial and temporal dynamics of social and environmental determinants of human health in urban systems.* Who gets sick and where do they live? What are the relative contributions of social versus environmental factors? What types of interventions are available and appropriate?

*Measures of health in different urban forms.* What contribution does urban pattern and social–ecological processes in urban environments make to the functionality of urban habitats? Can we identify the characteristics of dysfunctional and functional urban landscapes and incorporate this knowledge into better urban planning, design, construction and management?”

Table 3 identifies in more detail the elements of the urban built form and its transport systems and the geographical scale of the environmental stressor that are associated with human health, including hypothesizing on the dominant group in the population that might be especially exposed to the various stressors. For example, high concentrations of air pollutants in the ambient environment can result in breathing problems with human communities but the research challenge is to determine the spatial concentration or the geographical spread around transport systems.

Effective assessment of health-impact risk to exposed populations from air pollution, and other environmental stressors, is important for supporting decisions of the related detection, prevention, and correction efforts and therefore more research into estimating the geographical scale of exposure (from on the road in the case of traffic accidents to metropolitan air-sheds in the case of airborne pollutants) is needed. However, given the comments made in Section 2 about the need for a multi-disciplinary approach to the problem in Australia then inspiration for a collaborative research design could be drawn from INTARESE – a European research project [182] to develop a conceptual framework within which the latest scientific evidence across all the relevant environmental sectors, including transport, housing, agricultural land use, water management, household chemicals, waste management and climate, and disciplines is brought together as a basis for integrated assessment of both environmental and health impacts and risks. The aim is to build an integrated assessment methodology that can be applied to different stressors and environmental media, settings and locations (ambient, domestic, occupation) and stressors (chemicals, solid wastes, natural hazards, noise). By quantify and comparing environment and health risks (including international comparisons) then policy objectives and targets, and progress towards these policy targets can be established and communicated to key stakeholders. Some of the Australian groundwork in looking at, for example, the time-space patterns of road traffic pollutants has been accomplished at the Institute of Transport and Logistics Study, University of Sydney [183–186] but forging the links with health sciences remains to be undertaken.

In addition to the geographical extent of the environmental stressor is its time varying nature, both of which will influence the population exposure given their space-time trajectories through the “polluted spaces” including what is impacting on the residential space, is the difficult question of how to account for time. This “time” is in addition to the diurnal fluctuations in the environmental stressors mentioned above that have the potential to be measured with instruments or modeled (to form aggregated dose-response relationships). The difficult aspect of time to include in any analysis of the health of individuals is their history (conceptually from birth to death) – their cumulative time-space trajectories through the “historical” polluted places of their experience. One modeling approach to this highly complex problem with its longer-term migration and shorter term travel behaviors is to build on the generalized representation for a comprehensive urban and regional model [187] by explicitly incorporating the environmental stressors into the model.

Accounting for the various definitions of time is a research challenge. An initial start in this direction of dealing more explicitly with time has been made by the first author who wrote this article. The starting point is the quote, “as bodies age, the ability to defend against environmental stressors diminishes, and exposures over a life time can accelerate the aging process and trigger or exacerbate disease” [44]. An analysis of the data base extracting baby boomers from the Household, Income and Labour Dynamics in Australia (HILDA) is proposed .Respondents born between 1945 and 1962 – will be extracted and place of residence (that is, city location versus suburban location versus rural location) is used as a proxy variable for exposure to environmental pollutants, such as noise, motor vehicle emissions, particulate matter. The HILDA survey is a household-based panel study which began in 2001 with information on: households and family life; incomes and wealth; employment and unemployment / joblessness; and life satisfaction and wellbeing [188]. Where the trans-disciplinary approach becomes critical is in further research designs that bring together the experience, findings and strengths of the main-stream urban planning and transport researchers, the epidemiologists and health science experts and the key stakeholders from the policy sector.

## 4. Conclusions

The complexity of finding solutions to the impacts on public health of urbanization, climate change and specifically in the context of this article, transport, presents a major new challenge for sustainable development. Integrated solutions will require health care professionals, epidemiologists, engineers, environmental scientists, urban planners, designers and managers, policy specialists, economists and social scientists to grapple with working together in new ways, and to establish a common conceptual understanding. Team science working in a collaborative way within the trans-disciplinary framework described in this paper is a promising way forward but such teams require skilled leadership. All leaders are schooled in some form or other of management philosophy. However, those leaders who excel at generating and sustaining trust, who are supportive, democratic, inclusive, empowering, and are committed to encouraging cooperation and engaging the support of others by being generous in offering constructive feedback to colleagues will significantly enhance trans-disciplinary collaborations within the research team, within the research institute or university and amongst key stakeholders. Their encouragement of professional staff to pursue the trans-disciplinary thinking will bear fruits in creating innovation in research into cities, transport public health, sustainable policy, and environmental stewardship.

The literature cited in this paper demonstrates a richness across the themes of environmental stressors and their impacts on humans, on one hand, and of the processes of urbanization, transport and the environment. It does not claim to be comprehensive across all domains considered when searching for material but we hope the bibliography will be useful for other researchers to shape action-based, policy-relevant research projects. Our review of this literature concludes with there still being relatively little integration of the material. Repeated search strategies of data bases on key words such as “public health, urbanization, transport and environmental stressors” failed to identify much material. It is clear from the mainstream transport literature, and through the special interest groups of the World Conference on Transport Research Society that, within their environment group, transport and health is currently not on the main topic agenda. Similarly, although with some exceptions, the public health researchers have not connected adequately enough with the urban researchers. Champions are needed in both fields to advance trans-disciplinary research.

As a suggestion on research by way of bridging that gap, and recognizing that the core research team of a trans-disciplinary project must bring their our disciplinary skills and interests to the table, we suggest an ambitious challenge would be the explicit recognition of *time* in all phases from problem definition, through to reviewing existing knowledge on the research problem, especially disciplinary and inter-disciplinary conceptualizations and explanations, designing the research enquiry from research gaps; implementing the research enquiry, refining conceptual understandings and synthesizing data sets; and specifying types of interventions (with stakeholders) and their costs and benefits. One time dimension is represented by an individual’s life history: “as the body ages, the ability to defend against environmental stressors diminishes, and exposures over a life time can accelerate the aging process and trigger or exacerbate disease” [51]. We need to establish this life time exposure (in different places and locations) to the environmental stressors from transport systems and their cumulative effects on health and well-being. The methodology could be time-space geography but with the additional complexity of locations and magnitudes of environmental stressors mapped “through which people on their journeys” are exposed. The urban modelers – familiar with spatial interaction models using cross sectional data – need to turn their minds to the long-term dynamics of change as to how peoples’ travel patterns pass through “polluted” places as objects of investigation during their life histories as the body ages. The generalised urban and regional model presented by Sir Alan Wilson [197] is the point of departure for their potential contribution. We suggest that the issues of event (environmental stressor), time (from cradle to the grave), and place (locations, especially those with their own time-dependent variable of environmental stress) are the basis of a new type of epidemiological study.

## Figures and Tables

**Table 1:** Core Concepts and Domains of Health-Related Quality of Life.

**CONCEPTS AND DOMAINS**	**DEFINITIONS / INDICATORS**

**OPPORTUNITY**	
Social or cultural disadvantage Resilience	- Disadvantage because of health; stigma; societal reaction - Capacity for health; ability to withstand stress; physiological reserves

**HEALTH PERCEPTIONS**	
General health perceptions Satisfaction with health	- Self-rating of health; health concern/worry - Satisfaction with physical, psychological, social function

**FUNCTIONAL STATUS**	
**Social Function**	
Limitations in usual roles Integration Contact Intimacy and sexual function	- Acute or chronic limitations in usual social roles (major activities) of child, student, worker - Participation in the community - Interaction with others - Perceived feelings of closeness; sexual activity and/or problems
**Psychological Function** Affective Cognitive	- Psychological attitudes and behaviors, including distress and well-being - Alertness; disorientation; problems in reasoning
**Physical Function** Activity restrictions Fitness	- Acute or chronic reduction in physical activity, mobility, self-care, sleep, communication - Performance of activity with vigor and without excessive fatigue

**IMPAIRMENT**	
Symptoms/subjective complaints Signs Self-reported disease Physiological measures Tissue alterations Diagnoses	- Reports of physical and psychological symptoms, sensations, pain, health problems or feelings not directly observable - Physical examination; observable evidence of defect of abnormality - Patient listing of medical conditions or impairments - Laboratory data, records, and their clinical interpretation - Pathological evidence -Clinical judgments after “all the evidence”

**DEATH AND DURATION OF LIFE**	- Mortality; survival; years of life lost

**Table 2:** Mechanisms of Urban Development, Health Impacts and Potential Solutions.

**Mechanism**	**Health Impact**	**Management/Mitigation/Adaptation**
Economic Growth	Income and poverty Privatization of health services Globalization and city center expansion – heat island effect Centralization of specialist care	Tax redistributive policy Standards of public health care Tree planting; artificial moisture fine mist (World Expo, 2005) Government spatial planning
Suburbanization	Inequalities in access to medical services Isolation and stress	Government spatial planning Compact suburban developments with access on foot and cycling
Motorization	Car dependency and obesity Risk of road traffic accidents including pedestrians Exposure to traffic pollution on roads/parking lots	Promotion of healthy life-styles Traffic engineering - accident countermeasures & enforcement Engine technology; Transport policy - mode change to public transport
Road Congestion	Increasing vehicle emissions and exposure Increases drivers stress	Road pricing transport policy - mode change to public transport Priority for road-based public transport; public transport technologies
Energy Consumption	Greenhouse gas emissions, climate change and impacts Transport energy proportional to distance travelled; environmental load also trip distance related	Energy efficiency through technology (hydrogen) and green building codes and green transport modes Land use planning to make cities more compact and accessible
Environmental Load	Transport noise – annoyance and stress Lead in gasoline and children IQ Diesel particulate matter and respiratory problems	Engine technology; road pavement design; control speeds and traffic volumes Lead-free gasoline Engine/fuel technology; electrostatic precipitation filters in road tunnels

**Table 3:** Built Environment Impacts on Health and Geographical Scale of Problem.

**Interaction**	**Geographical Scale**	**Main Exposed Group**
Road and rail noise – sleep disturbance, annoyance, stress	Localized - noise attenuates with increasing distance	All groups in population, but greater impact on low – medium socio-economic status
Cyclists and ultra fine particulate matter inhalation	Localized – on road	Cyclists especially in morning peak
Aircraft noise - sleep disturbance, annoyance, stress	Area surrounding airport within 15–20 NEF	All groups with greater impact on low socio-economic status
Leaded petrol and child development	Localized to houses playgrounds on main roads, but note residual lead from paint within houses and gardens	Young children
Low density suburbs and stress	Suburban	Lower socio-economic status and those without access to automobile and reliable public transport
Car dependency and obesity	Urban, especially suburban	Adults and children
Transport emissions and air quality	Metropolitan air-shed	All groups, with dispersal modified by local meteorological conditions
Freeways and vehicle emissions and respiratory problems	within 500m – 1000m	All groups, especially young and elderly, and sub-groups
Diesel trucks, particulate matter and health	Near sea-ports, along major roads and truck routes	All groups but with a distinct socio-economic dimension
Accessibility and quality of life	Local neighborhood with emphasis on street design & pedestrian environments	All groups
Footpath design and standards pedestrian injuries	Localized around home to neighborhood activities	All groups but especially young and elderly
Poor dwelling design and layout and accidents in home	Household	Young children and elderly
